# Diagnosis of Newly Delivered Mothers for Periodontitis with a Novel Oral-Rinse aMMP-8 Point-of-Care Test in a Rural Malawian Population

**DOI:** 10.3390/diagnostics8030067

**Published:** 2018-09-15

**Authors:** Jussi M. Leppilahti, Ulla Harjunmaa, Jorma Järnstedt, Charles Mangani, Marcela Hernández, Taina Tervahartiala, Rodrigo Lopez, Ulla Ashorn, Per Ashorn, Dirk-Rolf Gieselmann, Timo Sorsa

**Affiliations:** 1 Department of Periodontology and Geriatric Dentistry, University of Oulu, Oulu 90014, Finland; jussi.leppilahti@student.oulu.fi; 2 Center for Child Health Research, University of Tampere, Tampere 33014, Finland; Harjunmaa.Ulla.G@student.uta.fi (U.H.); ulla.ashorn@uta.fi (U.A.); 3 Medical Imaging Centre, Department of Radiology, Tampere University Hospital, Tampere 33521, Finland; jorma.jarnstedt@PSHP.fi; 4 College of Medicine, University of Malawi, Private Bag 360, Chichiri, Blantyre, Malawi; cmangani@medcol.mw; 5 Laboratory of Periodontal Biology and Department of Pathology and Oral Medicine, Faculty of Dentistry, Universidad de Chile, Av. Sergio Livingstone Polhammer 943, Independencia, Santiago 8380492, Chile; mhernandezrios@gmail.com; 6 Department of Oral and Maxillofacial Diseases, Helsinki University Central Hospital, Stenbäckinkatu 9, PO BOX 100, Helsinki 00029, Finland; taina.tervahartiala@helsinki.fi; 7 Department of Dentistry and Oral Health, Aarhus University, Faculty of Health Sciences Aarhus University Vennelyst Boulevard 9, Aarhus 8000, Denmark; rlopez@dent.au.dk; 8 Department of Paediatrics, Tampere University Hospital Central Hospital PO BOX 2000, Tampere 33521, Finland; per.ashorn@uta.fi; 9 Institute for Molecular Diagnostics (IMOD), Bonner Str. 84, Solingen 42697, Germany; gieselmann@matrix-lab.de; 10 Division of Periodontology, Department of Dental Medicine, Karolinska Institute, Stockholm SE-171 77, Sweden

**Keywords:** alveolar bone loss, matrix metalloproteinase (MMP)-8, periapical infection, periodontitis, point-of-care testing, saliva

## Abstract

A novel qualitative point-of-care test of activated matrix metalloproteinase-8 (aMMP-8) using noninvasive oral rinse sampling procedures has been developed for the early detection of collagen breakdown indicating periodontal tissue destruction. The main object of this study was to assess the reliability of the test in a low-income setting to identify participants with history of periodontal destruction detected as alveolar bone loss (ABL) in radiographs. This cross-sectional study included 486 women who had recently delivered in rural Malawi. The aMMP-8 test and dental panoramic radiographs were taken within 48 h of delivery. The performance of the test in comparison to radiological examinations was tested by following the standards for reporting of diagnostic accuracy studies protocol (STARD) with respective statistical measures and 95% confidence intervals. From the 486 eligible participants, 461 mothers with complete data, aged from 15 to 46 years (mean 24.8, SD 6.0) were included in the analysis. ABL was identified in 116 of 461 participants. There was 56% agreement between the aMMP-8 test results and detected ABL (yes or no) in radiographs. Calculated sensitivity of the test was 80% (72–87%), specificity 48% (43–54%), positive predictive value 34% (31–37%), negative predictive value 88% (83–91%), positive likelihood ratio 1.55 (1.35–1.77), and negative likelihood ratio 0.41(0.28–0.60). The aMMP-8 test sensitivity and negative predictive value to identify the ABL cases were relatively high, but there was additionally a high rate of test-positive results in participants without ABL, especially in young mothers, leading to low overall agreement between the test results and radiological bone loss. Further longitudinal studies are needed to examine if the test positive subjects are in risk of future bone loss before the detectable signs of periodontitis in radiographs.

## 1. Introduction

Severe periodontitis, a destructive inflammatory condition of the tooth supportive tissues, is the sixth most prevalent disease globally, affecting approximately 11% of the population [1]. It has been estimated that in addition to enormous direct treatment costs, severe periodontitis causes productivity loss worth 54 billion US$ globally every year [2]. However, treatment of periodontitis is relatively simple and cost-effective if diagnosed at its early stages. Challenging enough, periodontitis is usually asymptomatic, i.e., a person who has the disease is not aware of it, and its traditional diagnostics require highly skilled dental professionals and equipment that are often not available, particularly in resource-poor settings [2,3,4].

A newly developed point-of-care test to diagnose periodontitis using mouth rinse, known as the aMMP-8 test, has recently been launched and a few validating studies documenting good diagnostic accuracy of the test are already available [5,6,7,8,9,10,11]. The test resembles a pregnancy test and it is easy to use. aMMP-8 test diagnostics are based on active matrix-metalloproteinase-8 (aMMP-8) level analysis by lateral flow immunoassay in mouth rinse and it provides information about the inflammatory burden of the periodontium [5,6,9,12,13,14]. In a previous study, aMMP-8 test differentiated adolescent patients having gingivitis or initial periodontitis from healthy participants with a good accuracy according to prior radiographic manifestations [5,6,7,8,9,10]. In addition, the positive test result was associated with the candidate susceptibility genes of periodontitis [6]. 

A main biological function of MMP-8 in the periodontium is to facilitate leukocyte, especially neutrophil granulocyte, migration from the circulation toward the periodontal sulcus by cleaving collagens and other extracellular matrix components [15]. In addition, other cell types (e.g., fibroblasts) can express MMP-8 after induction of inflammatory signals [16,17]. Abundant and noncontrolled MMP-8 expression, release, and activation together with other MMPs and proteinases are considered to cause inflammation-related tissue destruction in periodontitis and in other inflammatory diseases [13]. MMP-8 activation is facilitated by other MMPs and host-derived proteases and oxidative burst caused mainly by myeloperoxidase (MPO) released from neutrophils [18]. Bacteria-derived proteases, from, e.g., *Porphyromonas gingivalis* (gingipains) and *Treponema denticola*, can also activate MMPs [18]. Thus, the innovation from the diagnostic point of view is not to measure the total concentration of MMP-8 per se, but the active isoform of the enzyme [13,18]. The monoclonal antibody utilized in the aMMP-8–test is developed to target aMMP-8 indicating the vicious circle of the destructive inflammation process [13,17,18].

In a real-life situation, sources of oral inflammation and aMMP-8 are not restricted to periodontitis. In addition to periodontitis, the test might be able to detect periapical lesions (PAL) [13,19]. Periapical inflammation develops from endodontic infection, most commonly caused by deep caries lesions, and shares some etiopathogenic mechanisms with periodontitis. A recent study demonstrated that apical lesions can also modify the composition of oral fluids and can be acutely diagnosed through MMP-8 assessment in gingival crevicular fluid (GCF) [19].

This study is a part of larger MiTeeth study focused on assessment of maternal periapical infections and their association with infant birth size in a rural Malawian population. Maternal periapical infections were associated with a shorter pregnancy duration and intrauterine growth restriction in a previous study within the Malawian population [20]. Interestingly, the association was not found between periodontitis and the pregnancy outcomes, respectively [20]. The MiTeeth study was designed to obtain confirmation for the previous results within a different study sample from same area of Malawi. Coprevalence of periodontitis and periapical infections is common in this population [20], being also a challenge for study design and statistical analysis to extract interacting effects of periapical lesions and periodontitis exposure on the pregnancy outcomes. aMMP-8 tests were taken to gain information about the oral inflammatory burden and to complete radiological diagnosis of periapical lesions and periodontitis in a low-resource setting where clinical examination was not possible to conduct.

The aim of this presented study (a substudy of the larger MiTeeth study) was to assess the screening utility of the aMMP-8 test. The diagnosis of periodontitis is based on full-mouth continuous periodontal probing with a manual probe and detailed recording of clinical attachment levels, probing depths, visual plaque, and bleeding on probing from six sites of every tooth. The current golden standard method for screening periodontitis is clinical initial examination and determination of different periodontal screening indices (periodontal screening index (PSI) (in Germany) and periodontal screening and recording—PSR^®^ (established by the American Dental Association and the American Periodontal Association in 1992), which are derivatives of the community periodontal index (CPI) [21]. These indices are based on periodontal probing of every tooth at four sites. A site with the most severe disease defines the value of the index, and the index values are determined for each sextant of dental arch, making the procedure slightly faster in comparison to making the actual diagnosis [21,22,23,24]. In this study, periodontal probing was not possible to conduct and the testing of screening performance was done by comparing the aMMP-8 test with radiological diagnosis of alveolar bone loss indicating history of periodontitis. History of periodontitis is a known risk factor for future disease progression [25,26]. However, previous studies have found that the agreement between probing indices and radiological findings has not been very high [22,24]. The second aim was to study association between ABL, PAL, and the test results. To the best of our knowledge, this is the first study in which both ABL and PAL are taken into account when oral rinse aMMP-8 levels are studied. 

The study hypotheses were that aMMP-8 test results associate with ABL and possibly with PAL and that the test can differentiate the ABL cases. The respective null hypotheses were: (i) the aMMP-8 test cannot differentiate participants with and without ABL and (ii) aMMP-8 test results are not associated with ABL and PAL.

## 2. Results

### 2.1. Patient Characteristics of Included and Excluded Participants

Participants who had both the periodontitis rapid test and dental panoramic radiograph results available were included in the present analysis (*n* = 486). There were 25 mothers whose self-reported age was not known and were thus excluded from the regression analyses adjusted by maternal age and age stratifications. Participants characteristics for the included (*n* = 461) and excluded (*n* = 25) participants are described in Table 1. Body mass index (BMI), C-reactive protein (CRP), and haemoglobin (Hb) levels were measured to monitor maternal physiological condition after delivery. Thirty percent of mothers had CRP above the threshold of 40 mg/L, indicating bacterial infection. There were no significant differences between groups of included and excluded participants in CRP, BMI, or Hb levels. However, the frequency of human immunodeficiency virus (HIV) positivity and the prevalence of missing teeth and caries were slightly more common in the participants excluded due to the missing age information (Table 1).

### 2.2. Covariates/Cofactors of aMMP-8 Test Results

The potential covariates and cofactors of aMMP-8 test results are presented in Table 2. Maternal age was statistically significantly associated (*p* < 0.05) with the test results. There were no significant differences in CRP, BMI, or Hb levels, or in frequencies of HIV or malaria test results between participants with different aMMP-8 test results. From all listed oral-health-related variables, only ABL and PAL were significantly positively (*p* < 0.05) associated with aMMP-8 test results. The associations between the test results and ABL/PAL were further analysed with the multivariate regression models.

### 2.3. The Association between aMMP-8 Test Results and ABL and PAL

There was a statistically significant association between aMMP-8 test results and the extent of ABL and maternal age analysed with the generalized linear regression modelling (GLM). Results from the statistical model are given in Table 3. The positive test results (strong or weak) indicated approximately three times (strong positives, 3.5 teeth, 95% confidence interval (CI) 2.7–4.4), and two times (weak positives, 2.5 teeth, 95% CI 2.1–3.0) higher numbers of teeth with ABL compared with the negative test (1.2 teeth, 95% CI 1.0–1.5), when the model was adjusted by the fixed mean age of the mothers (24.8 years) (Table 3). 

The extent of PAL exhibited covariation with ABL in this data and is a potential modifier of aMMP-8 test results. The extent of PAL was significantly associated with the test results after the adjustment by age, but there were controversies in the pairwise comparisons: the weak positive test results associated significantly with the extent of PAL (*p* < 0.05), but the strong positives did not (*p* > 0.05) (Table 3).

The association of ABL and PAL with the aMMP-8 test result was further explored with the logistic regression model, in which the test result was the outcome measure and detected radiological findings of ABL and/or PAL was the explaining factor adjusted by the maternal age as the covariate. ABL alone (*p* < 0.05) or the combination of ABL and PAL (*p* < 0.001) associated significantly with the positive test results, but PAL alone did not associate with the positive results (Table 4). Maternal age was a confounder associating with ABL and PAL in the GLM described above, but could not explain significantly the test results in the logistic model, implying that age did not have any independent effect on MMP-8 levels.

### 2.4. Screening Utility of the aMMP-8 Test for Alveolar Bone Loss Cases

The screening utility of the aMMP-8 test to identify ABL cases referring to history of periodontitis was analysed by cross-tabulating the test results (negative or positive) and the dichotomous ABL-case variable (Table 5). The sensitivity of the test in the whole study population was 80% and specificity was 48%. Respectively, positive predictive values (PPV), i.e., rate of true positives from all positives, and negative predictive values (NPV), rate of true negatives, were 34% and 88%. Furthermore, the overall agreement of test results with the ABL cases, with at least two teeth with mild or more severe bone loss in radiographs, was 56%.

Respective measures were calculated also separately for stratified age groups: PPV ranged from 20% in the youngest age group up to 82% in the oldest group as the measures of NPV ranged from 93% to 67%, respectively. Sensitivity rates varied from 77% to 82% and specificity from 40% to 67% by the age groups. The agreement of the test results was from 54% among the youngest age group and in the group of 25–34-year-olds up to 77% in the group of the oldest mothers. 

**Figure 1:**
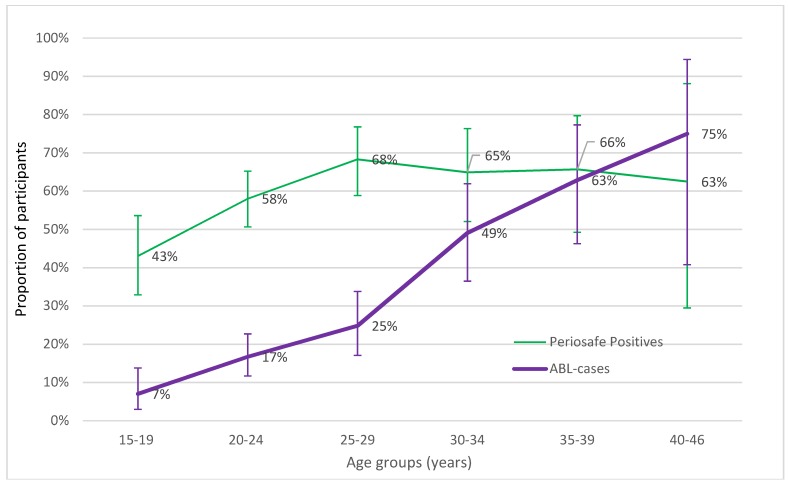
Proportion (%) of patients with aMMP-8 test positive result or ABL detected in radiographs. The proportion of aMMP-8 test positives is higher in young participants when compared to ABL.

The proportion of participants (crude prevalence) with the positive periodontitis screening result (aMMP-8 test-positive or radiological ABL case) was described further in smaller age groups with five-year intervals (Figure 1).

The proportion of aMMP-8 test positives increased from 43% in the youngest age group (15–19 years) up to 68% by the age of 25–29 years, being approximately the same in the older age groups (30–34, 35–39, and 40–46 years). In comparison, the proportion of radiological ABL cases increased from 7% in the youngest group up to 75% in the oldest participants (40–46 years old).

## 3. Discussion

Our study demonstrated the association between aMMP-8 test results and the extent of ABL in radiographs. There was no significant association between PAL and the test results if ABL and maternal age were taken into account in the statistical models, supporting the rejection of the null hypothesis related to ABL, but not to PAL. The test was sensitive enough to identify participants with ABL, but there was also a high rate of participants with positive test results, especially among the young participants, but no detectable ABL in radiographs, leading to a low overall agreement between the test results and the ABL cases, giving support for the first null hypothesis.

A weakness/limitation of this study is the lack of clinical periodontal data (periodontal probing measures). This topic is discussed with more detailed below. Another weakness is the unclear validity of self-reported or missing maternal age information. Strengths of the study include the large study sample size, comprehensive data collection methods, and completeness of the data.

Data stratification and the subgroup analysis revealed that the test agreement differed by participants′ age. Among younger mothers (from 15 to 34 years), the overall agreement was low, at around 50%, because of the large proportion of “false positive” results, i.e., positive test results without the bone loss in radiographs. In comparison, the agreement was 77% in the group of the oldest mothers (35–46 years), which is in line with previous validating studies with Finnish, German, Nigerian, and U.S. populations [5,6,7,8,9,10,11]. Because the lack of clinical periodontal probing measures, that would have enabled the detection of healthy, gingivitis, and initial periodontitis cases more accurately compared to X-ray data, it is not known if these young “false positive” mothers were true “false positives”, i.e., without bone loss and any periodontal inflammation, or actual “true positives” without bone loss but with significant periodontal inflammation with a risk of future bone loss. It is well known that radiographic findings of periodontal disease develop slowly, while the clinical signs are detectable earlier [22,24,28]. Ziebolz et al. compared PSI results to radiological diagnosis of periodontitis in dental panoramic X-rays. They found that in 47% of cases, the diagnosis was not converging, which is in line with our results. The proportion of aMMP-8 test positives reached the peak levels of 60–70% in five to ten years’ younger participants (25–29 years old) in comparison to the proportion of radiographic ABL cases (35–39 years old), suggesting that the test positivity eventually appears prior to bone loss. In fact, the test positivity could identify and reflect gingivitis and alert to so-called pre-periodontitis in participants without detectable ABL, thus making it more sensitive than identification based on radiographs solely, a finding also supported by previous studies [6,7,11]. 

It is also good to keep in mind that the closest clinical index corresponding to the aMMP-8 test is bleeding on probing (BOP). BOP is the golden standard to indicate the presence of active periodontal disease at the site and patient level [29,30]. Nevertheless, the BOP measurement is exposed to a subjective estimation of the clinician, and the oral fluid aMMP-8 detection could be seen in a more objective way to define the amount of inflammation in oral fluids. It has also been shown that the elevated levels of aMMP-8 in oral fluids precede and reflect future active periodontal degeneration [18,31,32]. However, further longitudinal studies are needed to examine if the test-positive subjects are at risk of future ABL before the detectable signs of periodontitis in radiographs and clinical outcomes arise.

There are not many epidemiological studies available describing the prevalence of periodontitis in the Malawian population [33]. A systematic review of the global burden of severe periodontitis in 1990–2010 gave an estimate of severe periodontitis prevalence to be 20.1% (9.2–35.9%) in Malawi [34]. In the review study, case definitions and inclusion criteria for severe periodontitis were based on clinically determined periodontal probing measures and periodontal indices, in comparison to radiographs and the aMMP-8 test in this study. In the present study, the ABL case definition included not only severe cases, but all cases, from mild localized to generalized severe alveolar bone loss [20]. It is also good to keep in mind that the present study was focused on the specific group of participants (relatively young females), excluding some risk groups of periodontitis (e.g., smokers, males). Thus, it is reasonable to assume that the prevalence of periodontitis was lower in the present study compared to the respective whole population in Mangochi district, Malawi. In an earlier study among a similar female Malawian (Mangochi district) population by Harjunmaa et al., the overall periodontitis prevalence was 31.9%, which is noticeably higher than in this study [21]. The difference is mostly explained by different diagnostic methods used in the earlier study based on both clinical and radiographic findings [21]. The previous study included also participants from more rural areas of Malawi, with higher prevalence of infections in comparison to people in the Mangochi area.

In this study, a significant proportion of participants were HIV-positive. HIV has been associated with periodontitis, and in theory, it could also have a modifying effect on MMP-8 levels [35]. All included HIV-positive participants had ongoing HIV medication in the present study. There were no significant associations observed between aMMP-8 and HIV test results in the descriptive analysis.

Oral inflammatory diseases such as marginal and periapical periodontitis share similar risk factors and forms with a genetically related cluster of patients also presenting systemic proinflammatory pathological manifestations such as cardiovascular diseases, stroke, diabetes, and adverse pregnancy outcomes [20,36,37,38,39]. Despite the widely reported associations between oral inflammatory diseases and systemic pathological conditions, it is not well-known whether oral inflammatory diseases are actual true risk factors of systemic diseases and what is the quality, extent, and severity of inflammation that matters if oral diseases are screened as a risk factor or marker in the perspective of systemic diseases. Whether it is an inflammatory burden indicated by a test such as the aMMP-8 test or a specific stage of periodontal/periapical disease defined by clinical/radiological examination, is an important question to answer in further studies, and addressed also by the MiTeeth research project (data not analyzed and published yet).

We conclude that the aMMP-8 test results correlate with the extent of alveolar bone loss in radiographs, but the overall agreement between aMMP-8 test results and the alveolar bone loss cases was low. Especially in young mothers, a significant proportion of participants had positive aMMP-8 test results without the bone loss. Further studies are needed to examine if the test-positive subjects without the bone loss have significant inflammation around periodontal tissues, explaining the positive results, and are at risk of future bone loss before the detectable signs of periodontitis in radiographs.

## 4. Materials and Methods

### 4.1. Design, Study Site, Participants, and Enrolment

This was a cross-sectional study, in which the utility of an oral rinse-based novel rapid point of care (POC) test for periodontitis to identify participants with radiographically detected ABL and PAL was evaluated.

This research is a substudy of a larger MiTeeth study that aimed to investigate the association between maternal oral health and infant birth size in rural Malawi, Southern Africa. Mothers who had given birth within 48 h were enrolled at the Mangochi District Hospital postnatal ward between April 2016 and October 2016. Participants for the present substudy were recruited between May and August 2016. Mangochi District Hospital serves an estimated population of 100,000. The district hospital catchment area is semiurban and it is serviced by all-weather roads. The population is mostly Chi-Yao speaking and subsists largely on farming and fishing. Malaria infection is endemic with seasonal fluctuations. Health services are provided through public facilities and are free at point of delivery. In this rural African population, oral infections are common, but except for tooth extractions, very little dental treatment is available.

**Figure 2:**
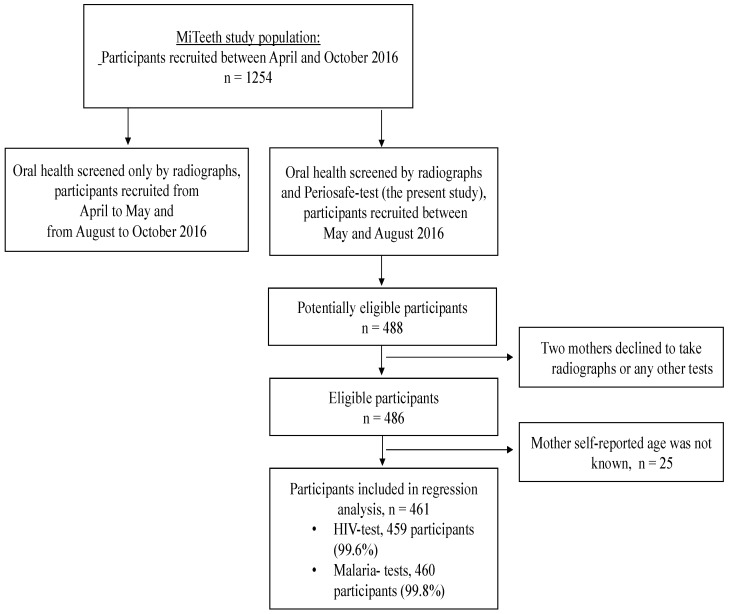
Schematic representation of participant flow.

Eligible participants were identified at the hospital postnatal ward. Women were briefed about the purpose of the study and its procedures and those wishing to participate went through an eligibility assessment. The inclusion criteria were: delivered a live infant at the hospital and signed or thumb-printed informed consent. The exclusion criteria were: age less than 15 years of age, multiple pregnancy, postnatal or maternal death prior to the data collection, full data cannot be collected due to the poor health condition of the mother or the infant, congenital malformation of the infant, data collected later than 48 h after delivery, regular smoker, participation in an intervention study during the pregnancy. Participant flow is described as detailed in Figure 2.

### 4.2. Data Collection

**Figure 3:**
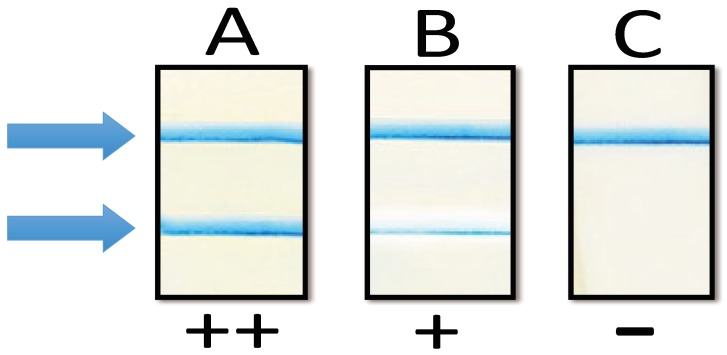
Example results of aMMP-8 point-of-care lateral flow immunotest. Two lines (indicated by arrows, ++, strong second line, lane **A**) indicate elevated risk for periodontitis and alveolar bone loss; even a thin second (indicated by arrows, +, weak one, lane **B**) line indicates elevated risk for periodontitis. One line indicates no risk for periodontitis (indicated by arrows, –, no second line, lane **C**).

The data collection was conducted at one visit within 48 h after delivery at the hospital postnatal ward and at the study clinic next to it. A study nurse interviewed the participants on their health history and relevant background information. Then, the periodontitis aMMP-8 POC test (PerioSafe^®^ PRO, Dentognostics GmbH, Jena, Germany) was performed in accordance to the manufacturer’s instructions. The test results were visually evaluated to be strong positive (++), weak positive (+), and negative (−), according the prespecified cutoff values of the test (example cases presented in Figure 3) [3].

After that, the nurse collected a finger prick blood test from the mother to diagnose C-reactive protein (CRP) and hemoglobin (Hb) concentrations and malaria status by rapid tests, and analysed them at the study site using test kits (QuickRead Go CRP + Hb, Orion Diagnostica, Espoo, Finland; Clearview^®^ Malaria Combo, Alere Inc, USA). The study nurse collected also information on the participants’ HIV status and treatment from the hospital records, or if missing, by asking the mother. An anthropometrist measured the length, weight, and mid-upper arm circumference (MUAC) of the mother. A dental therapist or a trained assistant conducted a structured interview on the oral health history of the mother and took a digital panoramic radiograph of her teeth and jaws (Planmega Proline XC, Planmeca, Finland).

An oral and maxillofacial radiologist (J.J.) and an experienced dentist (U.H.) blindly analysed the radiographs using digital imaging software (Planmega Romexis^TM^, Planmeca, Finland) at Tampere University Hospital Medical Imaging Center, Tampere, Finland. They recorded the number of teeth, caries lesions extending to the dentine or pulp, PAL, and marginal alveolar bone level at each tooth from the dentinoenamel junction to the bone margin, relative to the full root length (normal, initial, cervical, middle, or apical third) using structured forms. Initial ABL was determined as detectable “initial” signs without significant horizontal bone loss or vertical pockets in the radiological diagnosis. 

### 4.3. Ethics

Written or thumb-printed informed consent was taken from the participants at enrolment. Ethical approval was obtained from the College of Medicine Research and Ethics Committee, University of Malawi (No. P.09/15/1797), and the Ethics Committee of Pirkanmaa Hospital District, Finland (No. R16044, 3/2016). We enrolled only participants who signed or thumb-printed an informed consent. Participants who had dental diseases were provided complimentary dental treatment at Mangochi District Hospital dental clinic with resources provided by the research group.

### 4.4. Statistical Methods

#### 4.4.1. Descriptive Analysis of Study Population and Variable Definitions in Statistical Analysis

Different variables describing general (CRP, Hb, BMI, HIV, malaria) and oral health characteristics (self-reported oral hygiene behaviour, proportion of participants with ABL cases, caries, PAL, and missing teeth) of included (*n* = 461) and excluded participants (*n* = 25), and also aMMP-8 test result groups, were compared in tables by reporting means and 95% confidence intervals of continuous/count variables or, in the case of categorical (nominal) variables, the number of participants in each category and percentage proportion of it.

The number of teeth with ABL (initial, mild, moderate, or severe), PAL, caries, and missing teeth as ordinal count variables were used to describe the extent of different radiological findings. Categorical variables of ABL, PAL, caries, and missing teeth cases were used to report proportions of participants with the respective findings. The case definition for categorical variables was at least one tooth with the respective radiological finding, except for the ABL case. The ABL case definition was based on ABL extent and severity and was modified from the Eke et al. periodontitis case definition based on attachment loss measures [27]. The ABL case inclusion criterion was at least two teeth with mild or more severe ABL. Thus, mothers with initial ABL or localized mild or more severe ABL around only one tooth were not included into the ABL cases.

The used statistical units in the statistical analysis were the number of teeth in the analysis of associations between the aMMP-8 test and ABL/PAL and the number of participants in logistic regression analysis and the analysis of screening utility (see case definitions below).

#### 4.4.2. Association between the aMMP-8 Test and Extent of ABL or PAL

ABL and PAL associate with each other and share common risk factors, e.g., oral hygiene behaviour. They are both also strongly confounded by the age of the participant [40]. The association between the aMMP-8 test and extent of ABL and PAL were analysed using generalized regression modelling (GLM). The number of teeth with ABL (initial or more severe) or PAL was used as the dependent outcome variable indicating the extent of ABL or PAL. The number of teeth with a positive radiological finding was typical count data and was right-skewed. Negative binomial distribution with the log link was the chosen model type for analyses. The aMMP-8 test result was the explaining factor in the model. All regression models were adjusted by the participants’ age, the strong confounder of both ABL and PAL. Bonferroni correction for multiple hypothesis testing was utilized when post-hoc pairwise comparisons were reported. The statistical significance of effect estimates in the regression model and post-hoc pairwise comparisons were evaluated and reported with the *p*-value at 0.05 and 0.001 levels.

#### 4.4.3. Relative Size of Association between the aMMP-8 test and ABL in Comparison to PAL

Because ABL and PAL associate with and are covariates of each other, an additional logistic regression analysis was performed to analyse the relative size of the association between the aMMP-8 test result and ABL or PAL. In this analysis, dichotomous aMMP-8 test results, negative or positive (both weak and strong), were set as the outcome measure and the detected radiological findings of ABL and PAL were combined into a categorical (patient-level) explanatory variable in the model as follows: first, participants with no ABL or PAL; second, participants with ABL only; third, participants with PAL only; and fourth, participants with both ABL and PAL. The model was adjusted by the maternal age as a covariate. The categorical ABL case definition for the above-described radiological findings parameter was created, as described above, according to ABL extent and severity.

#### 4.4.4. Screening Utility of the aMMP-8 Test to Correctly Identify Periodontitis Referring to ABL Cases

The utility of the aMMP-8 test to identify participants with ABL findings was studied by cross-tabulating aMMP-8 test results and the ABL case variable. Sensitivity, specificity, positive (PPV) and negative predictive values (NPV), positive (LR+) and negative likelihood ratios (LR−), and the percentage of agreement between the test results and the ABL cases were reported by the tabulation. Cross-tabulation and respective calculations were performed using the whole study population and separately stratified by age groups (15–24, 25–34, 35–46 years). Age groups followed general recommendations for age stratifications in periodontal epidemiological studies [41]. 

## 5. Patents

Timo Sorsa is an inventor of U.S. patents 5652223, 5736341, 5866432, and 6143476, and Timo Sorsa and Dirk-Rolf Gieselmann are inventors of the WO 2015 128549 A-I patent, on the diagnostic use and method of analysis of MMPs and their inhibitors in oral fluids.

## Figures and Tables

**Table 1:** Characteristics of included and excluded participants.

Patient Characteristics			
		Included (*n* = 461)	Excluded (*n* = 25)
		Mean (95% CI)/Count (%)	Mean (95% CI)/Count (%)
General Health Characteristics			
Mother Age (years, self-reported)		24.8 (24.2–25.3)	-
BMI (kg/m^2^)		23.2 (22.9–23.5)	22.3 (21.1–23.4)
CRP (mg/L)		33.5 (31.0–36.0)	41.9 (25.2–58.6)
CRP above 40 mg/L		138 (30%)	8 (32%)
Haemoglobin Levels (g/L)		105 (103–106)	106 (98–114)
HIV test	Negative	411 (89%)	18 (72%)
	Positive	48 (10%)	7 (28%)
	Not Tested	2 (1%)	0
Malaria test	Negative	452 (98%)	25 (100%)
	Positive	8 (1.7%)	0
	Not Tested	1 (0.3%)	0
Oral Health Characteristics			
Tooth Cleaning (self-reported)	No daily basis cleaning	0	0
	Once a day	28 (6%)	2 (8%)
	Twice a day	433 (94%)	23 (92%)
Tooth Cleaning Method (self-reported)	Finger	16 (3%)	2 (8%)
	Toothbrush	445 (97%)	23 (92%)
aMMP-8 Test Results	Negative	189 (41%)	5 (20%)
	Weak positive	192 (42%)	15 (60%)
	Strong positive	80 (17%)	5 (20%)
Proportion of mothers with ABL (case) *		116 (25%)	8 (32%)
Proportion of mothers with caries		255 (55%)	19 (76%)
Proportion of mothers with PAL		127 (28%)	11 (44%)
Proportion of mothers with missing teeth		112 (24%)	11 (44%)

Body mass index (BMI); C-reactive protein (CRP); alveolar bone loss (ABL); periapical lesions (PAL); human immunodeficiency virus (HIV). * The case definition was based on radiological marginal bone loss detection and the case definition of Eke et al. [27] was modified to fit with radiological data.

**Table 2:** Potential covariates of aMMP-8 test results and alveolar bone loss.

Potential Covariates/ Cofactors of aMMP-8 Test		aMMP-8 Test Results		
		Negative (*n* = 189)	Weak Positive (*n* = 192)	Strong Positive (*n* = 80)
		Mean (95% CI)/Count (%)	Mean (95% CI)/ Count (%)	Mean (95% CI)/ Count (%)
General health-related variables				
Age (years, self-reported)		24 (23–25)	25 (24–26)	27 (25–28)
Haemoglobin (g/L)		105 (102–108)	103 (101–106)	106 (101–110)
BMI (kg/m^2^)		23.5 (23–24)	22.9 (22.5–23.3)	23.1 (22.3–23.8)
CRP (mg/L)		34.3 (30–38.5)	34.6 (30.8–38.3)	29 (24.3–33.7)
HIV	Positive	15 (8%)	24 (13%)	9 (11%)
	Negative	173 (92%)	168 (87%)	70 (88%)
	Not tested	1 (0.5%)	0	1 (1%)
Malaria	Positive	3 (1.5%)	5 (3%)	0
	Negative	185 (98%)	187 (97%)	80 (100%)
	Not tested	1 (0·5%)	0	0
Oral-health-related variables				
Tooth cleaning twice a day (self-reported)		178 (94%)	186 (97%)	69 (86%)
Tooth Cleaning Method (self-reported)	Finger	5 (3%)	4 (2%)	7 (9%)
	Toothbrush	184 (97%)	188 (98%)	73 (91%)
Number of teeth with ABL		1.4 (1.0–1.8)	3.0 (2.4–3.6)	4.9 (3.7–6.2)
Proportion of mothers with ABL (case)		23 (12%)	58 (30%)	35 (44%)
Number of teeth with caries		1.6 (1.3–1.9)	2.2 (1.8–2–7)	1.9 (1–3–2–4)
Proportion of mothers with caries		96 (51%)	114 (59%)	45 (56%)
Number of teeth with PAL		0.4 (0.2–0.5)	0.6 (0.5–0.8)	0.7 (0.4–0.9)
Proportion of mothers with PAL		43 (23%)	55 (29%)	29 (36%)
Number of missing teeth		0.4 (0.3–0.5)	0.6 (0.4–0.8)	0.7 (0.3–1.0)
Proportion of mothers with missing teeth		45 (24%)	43 (22%)	24 (30%)

**Table 3:** Association between aMMP-8 test results and marginal alveolar bone loss or periapical lesions.

Outcome	Models *	aMMP-8 Test Results			
		Negative (*n* = 189)	Weak Positive (*n* = 192)	Strong Positive (*n* = 80)	*p*-Value (Test of Model Effects)
Number of teeth with ABL	Adjusted marginal means	1.3 (1.0–1.5)	2.5 (2.1–3.0)	3.5 (2.7–4.4)	<0.001
	Pairwise comparison (mean difference)	-	1.3 (0.7–1.9)	2.2 (1.1–3.3)	
	*p*-value(pairwise comparison)	-	<0.001	<0.001	
Number of teeth with PAL	Adjusted marginal means	0.3 (0.3–0.5)	0.6 (0.5–0.7)	0.5 (0.4–0.8)	<0.05
	Pairwise comparison (mean difference)	-	0.2 (0.0–0.4)	0.2 (−0.1–0.5)	
	*p*-value (pairwise comparison)	-	<0.05	>0.05	

Alveolar bone loss (ABL), periapical lesion (PAL); * Models are adjusted by fixed estimate of mean mother age: 24.8 years.

**Table 4:** Logistic regression model on association between the radiological findings and aMMP-8 test results adjusted by mother age.

Outcome: aMMP-8 Test Result *		Adjusted Model (*n* = 461)	
Explaining factors and covariates		Odds ratio (95% CI)	*p*-Value
Radiological finding	No ABL or PAL (*n* = 270)	1 (ref)	<0.001
	ABL but no PAL (*n* = 64)	2.7 (1.4–5.1)	<0.05
	PAL but no ABL (*n* = 75)	0.98 (0.59–1.7)	Ns
	Both ABL and PAL (*n* = 52)	5.7 (2.4–13.4)	<0.001
Mother age		1.00 (0.99–1.01)	Ns

Alveolar bone loss (ABL), periapical lesion (PAL), statistically nonsignificant (ns); * Outcome: Dichotomous aMMP-8 test result (negative vs. positive, both weak and strong).

**Table 5:** Cross tabulation of aMMP-8 test results and the dichotomous ABL-case variable.

		aMMP-8 Test								
Age Group	ABL Case	Negative	Positive	Measures of Diagnostic Screening Utility						
		Count	Count	Sens.	Spec.	PPV	NPV	LR+	LR–	Accuracy
**15–24 years**	No	114	111							
	Yes	8	27	77 (60–90)%	51 (44–57)%	20 (16–23)%	93 (88–96)%	1.56 (1.25–1.96)	0.45 (0.24–0.84)	54%
**25–34 years**	No	42	63							
	Yes	10	43	81 (68–91)%	40 (31–50)%	41 (36–46)%	81 (70–89)%	1.35 (1.10–1.66)	0.47 (0·26–0.86)	54%
**35–46 years**	No	10	5							
	Yes	5	23	82 (63–94)%	67 (38–88)%	82 (69–91)%	67 (46–83)%	2.46 (1.18–5.15)	0.27 (0.11–0.64)	77%
**Total**	No	166	179							
	Yes	23	93	80 (72–87)%	48 (42–54)%	34 (31–37)%	88 (83–91)%	1.55 (1.35–1.77)	0.41 (0.28–0.60)	56%

Alveolar bone loss (ABL), sensitivity (sens.), specificity (spec.), positive predictive value (PPV), negative predictive value (NPV), positive likelihood ratio (LR+), negative likelihood ratio (LR−).
